# Absorption and Tensility of Bioactive Sutures Prepared for Cell Transplantation

**DOI:** 10.3390/ma6020544

**Published:** 2013-02-15

**Authors:** Dénes B. Horváthy, Gabriella Vácz, Tamás Szabó, Károly Renner, Kinga Vajda, Balázs Sándor, Zsombor Lacza

**Affiliations:** 1 Institute of Human Physiology and Clinical Experimental Research, Semmelweis University, Tűzoltó 37-47, Budapest H-1094, Hungary; E-Mails: vaczgabi@gmail.com (G.V.); vajdakinga89@gmail.com (K.V.); sandorbm@gmail.com (B.S.); zlacza@mac.com (Z.L.); 2 Department of Orthopedics, Semmelweis University, Karolina 27, Budapest H-1113, Hungary; 3 Department of Interfaces and Surface Modification, Institute of Materials and Environmental Chemistry, Hungarian Academy of Sciences, Pusztaszeri 59-67, Budapest H-1025, Hungary; E-Mails: szabo.tamas@chemres.hu (T.S.); krenner@mail.bme.hu (K.R.); 4 Department of Physical Chemistry and Material Science, Budapest University of Technology and Economics, Műegyetem 3, Budapest H-1111, Hungary

**Keywords:** absorbable suture, stem cell, cell therapy, tensile properties, absorption

## Abstract

Biodegradable scaffolds are widely used to transplant stem cells into various tissues. Recent studies showed that living stem cells can be attached to the surface of absorbable sutures *in vitro*. Soaking the absorbable material polyglactin in a cell culture medium and thereby creating a stem cell biofilm on its surface may initiate the absorption process even before implantation; therefore, the physicochemical properties of the suture may be compromised *in vivo*. We found that pre-incubation of sutures in cell culture media *in vitro* results in tensile strength reduction and faster suture absorption in a rat model of muscle injury. Shorter incubation times of up to 48 h do not influence absorption or tensility; therefore, it is advisable to limit incubation times to two days for polyglactin-based cell delivery protocols.

## 1. Introduction

Suture selection in clinical practice is determined by the host tissue and other features, such as the tensile strength, absorption, handling and biocompatibility of the suture. Insufficient wound healing that is caused by failure of the closing material. Therefore, suture strength changes that are caused by the biological environment warrants investigation. Freudenberg has shown that incubating various absorbable closing materials in different body fluids and pH buffers *in vitro* significantly decreases the tensile strength of sutures [1]. These differences have been supported by *in vivo* experiments: Karabulut tested the tensile strength and absorption of seven suture materials in rats and suggested that suture and host tissue characteristics should be strongly considered before the suture application [2].

Recent studies have investigated absorbable sutures as a potential scaffold for stem cell transplantation [3,4,5]. In our previous study, we also showed that surgical sutures coated with proteins and cells could function as a stem cell delivery system [6]. In addition, Yao *et al.* also tested cell coated sutures *in vivo* by transecting and repairing the Achilles tendon in rats [7]. Even though suture-based cell delivery seems to be a viable way to transplant stem cells into soft tissues, there is no scientific data investigating the biomechanical features of the sutures after the cell-coating preparation steps. Diminished tensile properties even with the presence of exogenous stem cells could result in insufficient healing in tissues like tendon, where good initial strength is needed for tissue apposition. Our hypothesis in the present investigation is that extended incubation under standard cell culture conditions and freeze-drying may compromise the original features of the sutures. Therefore, we tested the *in vivo* absorption and tensility of our cell-coated bioactive sutures that were subjected to cell culture conditions before implantation.

## 2. Results

### 2.1. *In Vivo* Absorption

**Figure 1:**
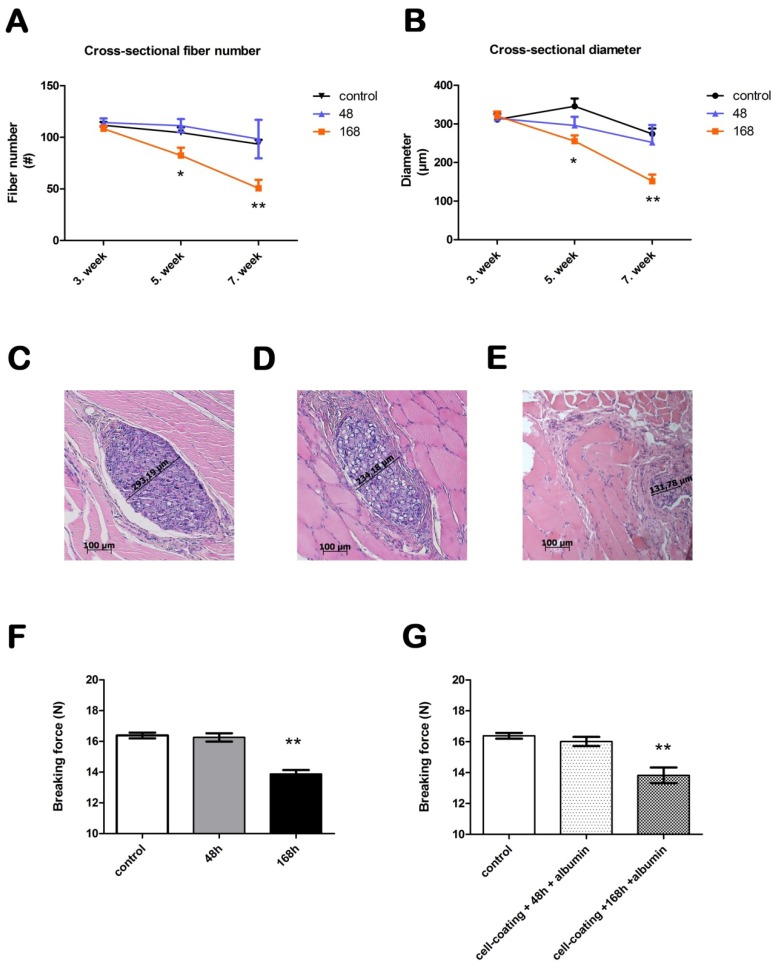
Absorption and tensility of bioactive sutures prepared for cell transplantation. Panel (**A**) shows the cross-sectional fiber number and Panel (**B**) shows the cross-sectional diameter *in vivo*. Panels (**C**–**E**) show the histology of the skeletal muscle after 7 weeks (hematoxylin and eosin). The cross-sectional diameter of a control suture can be seen in panel (**C**). Panels (**D**) and (**E**) show sutures that were incubated for 48 and 168 h *in vitro*, respectively. The images were taken at 10× magnification. The scale bar represents 100 µm. Panel (**F**) shows the breaking force of sutures after 48 and 168 h of incubation. Panel (**G**) shows the breaking force of the sutures after cell coating and incubation for 48 and 168 h.

Absorption was determined using the cross-sectional diameter and fiber number of the sutures in the muscle tissue. The albumin coating caused no significant difference in the *in vivo* absorption at any time point compared with the control sutures. After three weeks of *in vivo* incubation, we observed no significant differences between the control, 48 and 168 h incubated sutures in either diameter or fiber number. After five weeks of absorption, however, the suture that was incubated for 168 h had a significantly smaller diameter and lower fiber number (p < 0.05). The diameter of the control suture was 345.9 ± 20.0 µm, while the suture that was incubated for 168 h had a diameter of 255.5 ± 14.9 µm (Figure 1). The fiber number of the control suture was 104.6 ± 5.1 after five weeks, while the fiber number of the suture that was incubated for 168 h was 82.4 ± 7.5 (Figure 1). These differences were more pronounced at seven weeks (p < 0.0001): the suture incubated for 168 h had an even lower cross-sectional diameter (151.7 ± 16.9 µm) and fiber number (50.74 ± 8.0) compared to the control suture (274.2 ± 14.0 µm and 93.4 ± 4.2, respectively). The suture that was incubated for 48 h did not significantly differ from the control suture at any time point in the cross-sectional analysis. At five weeks, the diameter was 296.3 ± 22.0 µm, and the fiber number was 111.3 ± 6.4 (Control suture at five weeks: 345.9 ± 20 µm; 104.6 ± 5.1). After 2 additional weeks of absorption, the diameter decreased to 252.1 ± 45.1 µm, and the fiber number was 98.33 ± 18.7 (Control suture at seven weeks: 274.2 ± 14.0 µm; 93.4 ± 4.2).

### 2.2. Suture Tensility

The breaking force of the control sutures was 16.38 ± 0.2 N, to which we compared the experimental sutures after they were freeze-dried with albumin and incubated in a cell culture medium (Figure 1). After 48 h of incubation, the breaking force of the sutures, with and without albumin coating, did not change significantly compared to the control sutures (with albumin, 15.31 ± 0.3 N; without albumin, 16.26 N ± 0.3). Conversely, the breaking force of the sutures that were incubated for 168 h was reduced by 16%–19% (with albumin, 13.34 ± 0.3 N; without albumin, 13.88 ± 0.3 N, p < 0.0001). We also tested sutures with viable cells attached to the surface. The cell-coated sutures were incubated for 48 and 168 h as well. After 48 h the tensile properties were comparable to that of the control sutures (16.02 ± 0.3 N). After 168 h of incubation the breaking force of cell coated sutures decreased significantly (13.82 ± 0.5 N). This reduction was similar to those incubated for 168 h without treatment.

### 2.3 Inflammatory Response

**Figure 2:**
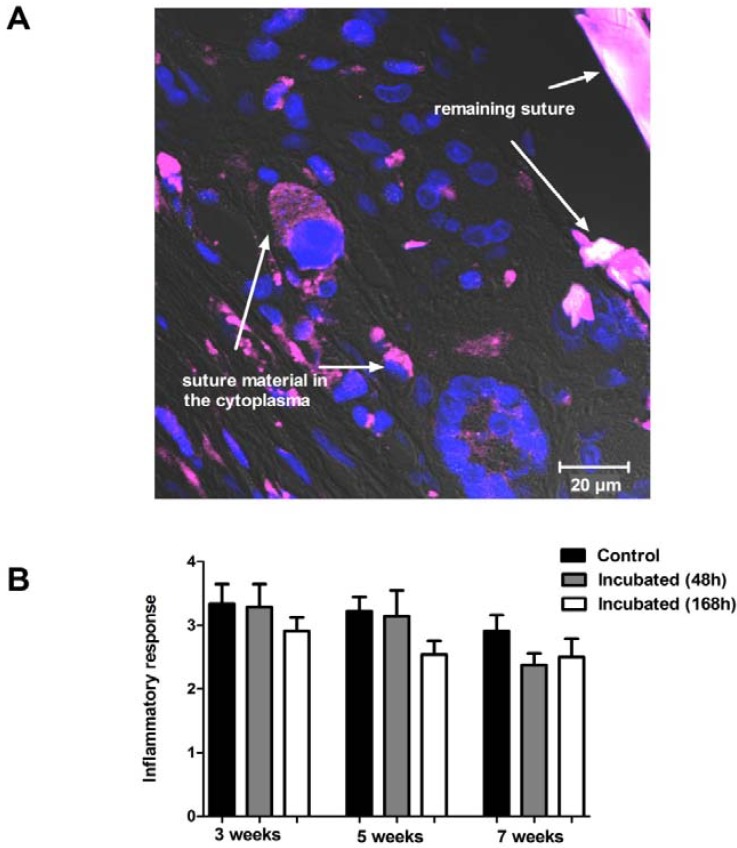
Inflammatory response and phagocytosis of implanted suture material. Panel (**A**) shows a section from skeletal muscle with the remaining suture material (purple) 5 weeks after the implantation (63×). Blue color represents the nuclei of the cells. Degraded suture material can be seen in the cytoplasm of the surrounding cells. Panel (**B**) shows the inflammatory reaction to the control and to the 48 or 168 h pre-incubated suture material after 3, 5, or 7 weeks of implantation. No significant difference can be observed between the groups at any time point.

Inflammatory response was seen around every suture cross section in every time period. There was no significance between the groups in the severity of the inflammatory response, but incubated sutures showed lower reaction compared to the control sutures (Figure 2). At a morphological level it can be seen that poly-ester fragments are phagocyted by inflammatory cells in the tissue five weeks after implantation (Figure 2).

## 3. Discussion

In the present study, we observed that a week-long incubation under standard cell culture conditions significantly decreased the breaking force of polyglactin closing materials by 16%–19% and also reduced the absorption time. Conversely, when the incubation time lasted for only two days, the aforementioned characteristics did not change compared to the control sutures. Because absorbable sutures are partly degraded by cellular activity [1], it is important that their tensility is unaffected by attached bone marrow-derived stem cells and is comparable to that of uncoated sutures. We found that attached stem cells do not change the breaking force of sutures *in vitro*; moreover, remaining poly-ester fragments are phagocyted *in vivo* by inflammatory cells. From this data we conclude that the reason for decreased tensility and faster absorption is spontaneous hydrolysis *in vitro*. In addition, biodegradable scaffolds are widely used to transplant stem cells into various tissues. The advantage of our technique is that it eliminates the need for an extra scaffold because the cell-coated sutures merge cell transplantation with the surgeon’s first therapeutic choice in situations that require tissue apposition.

In our previous study, we created confluent cell layer on the surface of the suture after 168 h of incubation. By lowering the incubation time to 48 h the cell number is about one-third (nucleic fraction 154 ± 35 µm^3^ *versus* 60 ± 20 µm^3^) [6]. Therefore, if the sutures are to be used only as cell-delivery systems in which a lower strength is sufficient to keep the tissues together, then faster absorption could be advantageous. In these situations, a longer incubation time with significantly higher cell numbers can be used to ultimately make cell therapy more effective. In tendon tissue or skeletal muscle, however, suitable initial and well-retained tensile properties are needed. Therefore, the surgeon cannot risk insufficient wound healing by using sutures with compromised physical and absorptive properties [8]. Note that in tendon tissue and skeletal muscle, the cellular turnover is rather low; therefore, a fine suture with a smaller number of attached cells could support the regenerative processes. In these situations, a two-day long incubation would be an appropriate choice for a suture-based cell delivery system.

## 4. Experimental Methods

### 4.1. Animals

Male Wistar rats weighing 250–300 g were used. The animals were maintained on lab chow and tap water ad lib with 12 h day/night cycle in the animal facility of the Institute of Human Physiology and Clinical Experimental Research in Budapest. The investigation was approved by the local Animal Research Committee according to the guidelines for animal experimentation.

### 4.2. Suture Preparation

Braided, absorbable 5-0 polyglactin sutures (Vicryl, Johnson *&* Johnson, Janssen-Cilag Ltd., Hungary) were used in our study. Albumin was freeze-dried on the surface of the sutures, and the sutures were incubated in modified Dulbecco’s DMEM at 37 °C for 48 or 168 h. The sutures that lacked an albumin coat were also incubated. The cell-coated sutures were created as described in our previous work. Briefly, the albumin-coated sutures were incubated for 48 or 168 h with human bone marrow-derived mesenchymal stem cells (100,000 cells/cm) under standard cell culture conditions. Untreated sutures served as the negative controls [6].

### 4.3. *In Vivo* Absorption

The incubated sutures were examined *in vivo*. Under pentobarbital (0.15 mL/100 g, 1:10 Nembutal) anesthesia, the incubated and control sutures were placed into the gluteal and triceps surae muscles of male Wistar rats. The animals were sacrificed, and the muscle samples were harvested at three, five and seven weeks. Paraffin sections from the formalin-fixed tissues were stained with hematoxylin and eosin. The sections were blindly analyzed by calculating their cross-sectional diameter and counting the remaining fibers along the sutures in the muscle tissue. Only true cross-sectional sutures were examined.

### 4.4. Suture Tensility

The tensility of the prepared sutures was assessed by an Instron 5566 mechanical testing machine (Instron, Norwood, MA, USA). The breaking force was calculated at 10 mm/min cross-head speed; the gauge length was 40 mm, and the temperature and relative humidity were 23 °C and 50%, respectively. The sutures were reeled onto columns to prevent slippage.

### 4.5. Inflammatory Response

Inflammatory response around the remaining sutures was analyzed in a 1–5 scale in a blinded fashion from hematoxylin stained sections, where 5 was severe inflammation and one represented no inflammation. Degradation process was investigated with laser confocal microscopy (Zeiss LSM 510 META, Carl Zeiss, Jena, Germany); suture material showed autofluorescence at 488 nm. Hoechst 33342 (Molecular Probes, #H3570) was applied to counterstain the cell nuclei

### 4.6. Statistical Analysis

All of the values are reported as the means ± SEM. The statistical analysis was performed using one-way ANOVA and Dunnett’s multiple comparison test using the GraphPad Prism statistical software (* p < 0.05, ** p < 0.0001).

## 5. Conclusions

In conclusion, the surface of absorbable sutures can function as a vehicle for stem cell implantation, but it is important to know that a week-long pre-incubation of the sutures decreases the breaking force and induces faster absorption *in vivo*. This drawback can be eliminated by reducing the *in vitro* incubation time to 48 h; therefore, it is advisable to develop shortened culture protocols for suture-based cell delivery.
